# Stabilized O3‐Type Layered Sodium Oxides with Enhanced Rate Performance and Cycling Stability by Dual‐Site Ti^4+^/K^+^ Substitution

**DOI:** 10.1002/advs.202304067

**Published:** 2023-09-26

**Authors:** Lin‐Rong Wu, Yu‐Han Zhang, Zhen Wu, Jinlv Tian, Haorui Wang, Haijun Zhao, Shoudong Xu, Liang Chen, Xiaochuan Duan, Ding Zhang, Huijuan Guo, Ya You, Zhi Zhu

**Affiliations:** ^1^ College of Chemical Engineering and Technology Taiyuan University of Technology 79 Yingze West Street Taiyuan 030024 P. R. China; ^2^ Qingdao Industrial Energy Storage Research Institute Qingdao Institute of Bioenergy and Bioprocess Technology Chinese Academy of Sciences Qingdao 266101 P. R. China; ^3^ School of Future Technology University of Chinese Academy of Sciences Beijing 100049 P. R. China; ^4^ State Key Laboratory for Mechanical Behavior of Materials Xi'an Jiaotong University Xi'an 710049 P.R. China; ^5^ College of Chemistry Taiyuan University of Technology 79 Yingze West Street Taiyuan 030024 P. R. China; ^6^ School of Chemical Engineering and Pharmacy Wuhan Institute of Technology Wuhan 430205 P. R. China; ^7^ State Key Laboratory of Advanced Technology for Materials Synthesis and Processing Wuhan University of Technology Wuhan 430070 P. R. China; ^8^ International School of Materials Science and Engineering School of Materials and Microelectronics Wuhan University of Technology Wuhan 430070 P. R. China; ^9^ School of Energy and Environment Southeast University Nanjing 211189 P. R. China

**Keywords:** K/Ti co‐doping, NaNi_0.5_Mn_0.5_O_2_, O3‐oxide cathode, reversible phase transition, sodium‐ion batteries

## Abstract

High‐capacity O3‐type layered sodium oxides are considered one of the most promising cathode materials for the next generation of Na‐ion batteries (NIBs). However, these cathodes usually suffer from low high‐rate capacity and poor cycling stability due to structure deformation, native air sensitivity, and interfacial side reactions. Herein, a multi‐site substituted strategy is employed to enhance the stability of O3‐type NaNi_0.5_Mn_0.5_O_2_. Simulations indicate that the Ti substitution decreases the charge density of Ni ions and improves the antioxidative capability of the material. In addition, the synergistic effect of K^+^ and Ti^4+^ significantly reduces the formation energy of Na^+^ vacancy and delivers an ultra‐low lattice strain during the repeated Na^+^ extraction/insertion. In situ characterizations verify that the complicated phase transformation is mitigated during the charge/discharge process, resulting in greatly improved structure stability. The co‐substituted cathode delivers a high‐rate capacity of 97 mAh g^−1^ at 5 C and excellent capacity retention of 81% after 400 cycles at 0.5 C. The full cell paired with commercial hard carbon anode also exhibits high capacity and long cycling life. This dual‐ion substitution strategy will provide a universal approach for the new rational design of high‐capacity cathode materials for NIBs.

## Graphical abstract

This work adopts a dual‐site substitution strategy to enhance the bulk and interfacial stability of O3‐NaNi_0.5_Mn_0.5_O_2_ cathode. The prepared cathode delivers high stability during repeated electrochemical cycles. The introduction of K/Ti can effectively suppress multiple phase transitions, reduce the Na^+^ vacancy formation energy, induce the formation of a NaF‐rich, thin but stable CEI layer, and improve air stability.

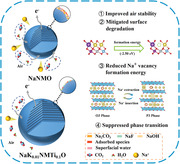

## 1 Introduction

Na‐ion batteries (NIBs) have been considered as the potential alternative to Li‐ion batteries (LIBs) in large‐scale energy storage devices because of abundant reserves and low cost.^[^ ^1^ ^]^ Similar to LIBs, cathode materials are crucial to the operating voltage, energy density, and even cycle life of NIBs.^[^ ^2^ ^]^ The layered transition metal (TM) oxides Na*_x_* TMO_2_ have been widely studied as cathodes for NIBs due to their obvious advantages including a simple synthesis process and attractive electrochemical properties.^[^ ^3^ ^]^ P2 phase (*x* < 0.8, Na^+^ occupies trigonal prismatic sites, O sheets stack in an ABBA sequence) and O3 phase (0.8 ≤ *x* ≤ 1, Na^+^ occupies octahedral sites, O sheets stack in an ABCABC sequence) are representative cathode structures, and the structure can be predicted by modulating the Na content and cation potential *Φ*.^[^ ^4^ ^]^ With higher reversible capacity relative to the Na‐poor P2‐cathodes in the same voltage window, the Na‐rich O3‐cathodes have been studied as the candidate cathode materials matched with sodium‐free anodes to assemble full cells.^[^ ^5^ ^]^

Based on Ni^2+^/Ni^3+^/Ni^4+^ redox, the typical O3‐type cathode NaNi_0.5_Mn_0.5_O_2_ delivers a large reversible capacity and high average discharge voltage.^[^ ^1^, ^6^ ^]^ However, their large‐scale application is suffering from some severe challenges. For example, the detrimental multiphase transformations and unavoidable electrolyte erosion during Na^+^ de/intercalation process are prone to rapid capacity decay and deteriorated rate capability of NaNi_0.5_Mn_0.5_O_2_.^[^ ^7^ ^]^ Further, most O3‐type cathodes are sensitive to moisture and susceptible to Na^+^ release via Na^+^/H^+^ exchange or TM oxidation, thus inducing severe degradation of the electrochemical performance.^[^ ^8^ ^]^

To address these issues, the conventional strategy is to adopt surface coating and elemental substitution.^[^ ^9^ ^]^ Usually, the surface coating has been considered a direct method to protect the cathode from electrolyte erosion; however, it cannot modulate the unfavorable phase transition of the bulk phase. Chemical substitution has been demonstrated to improve electrochemical performance by optimizing the bulk structure and surface interface properties of the cathode material. For example, introducing small amounts of Mg^2+^ into the TMO_2_ slabs can effectively suppress irreversible multiphase transitions to achieve high structural stability.^[^ ^10^ ^]^ It has proven to be essential to investigate the mechanism of multi‐site doping to synergistically optimize the electrochemical performance of cathode, such as Cu/Ti co‐substituted NaNi_0.45_Cu_0.05_Mn_0.4_Ti_0.1_O_2_,^[^ ^11^ ^]^ Fe/Mg co‐substituted NaNi_0.35_Fe_0.2_Mg_0.05_Mn_0.4_ O_2_,^[^ ^12^ ^]^ and Co/Ti co‐doped NaNi_0.4_Mn_0.25_Ti_0.3_Co_0.05_O_2_,^[^ ^13^ ^]^ etc. The above‐doped transition metal ions are generally introduced into the TM layer (a small amount of Mg^2+^ may occupy the Na layer) to suppress the unfavorable multiphase transition. Some pioneers have confirmed that reasonable Na site substitution is also important to enhance structural stability and Na^+^ diffusion kinetics. In this case, an optimal dual‐site substitution combining the advantages of both Na layer and TM layer substitutions may obtain excellent electrochemical properties.

Herein, K^+^ and Ti^4+^ have attracted our attention and tend to partially replace Na^+^ and Mn^4+^ according to their ionic radius and elemental valence states. First, it is demonstrated that Ti^4+^ can efficiently expand the solid solution reaction region, improve air stability, and inhibit electrolyte erosion.^[^ ^11^, ^14^ ^]^ Furthermore, the introduction of K^+^ facilitates (de) sodiation and stabilizes the structure due to the larger ionic radius and lower electronegativity than Na^+^.^[^ ^15^ ^]^ Therefore, taking full advantage of both elements offers the possibility of designing high‐specific energy and long‐life cathode materials.

## 2 Results and Discussion

### 2.1 Microstructure and Composition Characterization

**Figure 1:**
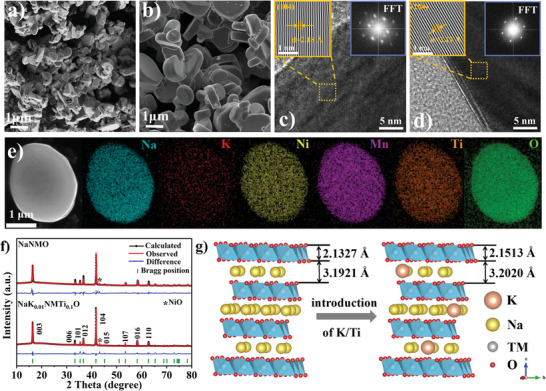
SEM images of a) NaNMO and b) NaK_0.01_NMTi_0.1_O. HR‐TEM images with FFT patterns as an insert of c) NaNMO and d) NaK_0.01_NMTi_0.1_O. e) EDS mappings of NaK_0.01_NMTi_0.1_O. f) XRD patterns and Rietveld refinement plots of NaNMO and NaK_0.01_NMTi_0.1_O. g) Schematic diagrams of crystal structure model of NaNMO and NaK_0.01_NM Ti_0.1_O.

As shown in **Figure** **1**a and Figure S1 (Supporting Information), the NaNi_0.5_Mn_0.5_O_2_ sample (referred to as NaNMO) delivers a plate‐like morphology with an average particle size of ≈400 nm and thickness of ≈200 nm. However, though the synthesis method and sintering procedure are identical to those of NaNMO, the particle size of the Na_0.99_K_0.01_Ni_0.5_Mn_0.4_Ti_0.1_O_2_ sample (referred to as NaK_0.01_NMTi_0.1_O) increases significantly to ≈1 µm, which is mainly due to that Ti can facilitate the crystal growth during sintering (Figure 1b; Figure S2, Supporting Information).^[^ ^16^ ^]^ The formation of large‐sized single crystals and the reduced specific surface area tends to cause a decreased contact area with air, thus inhibiting the production of residual bases on the surface.^[^ ^11^ ^]^ The interplanar distances between adjacent lattice stripes in the HRTEM pattern is 2.19 Å, in accordance with the *d*‐spacing value in the (104) plane calculated from the Bragg equation (2*d* sin*θ* = n*λ*) (Figure 1d). EDS shows that all elements are uniformly distributed on the sample surface (Figure 1e; Figure S3, Supporting Information). The XRD patterns of the four samples are considered as O3‐type *α*‐NaFeO_2_ layered structures with rhombohedral *R‐3m* space group (Figure S4, Supporting Information).^[^ ^11^, ^17^ ^]^ The observed and calculated patterns of NaNMO and NaK_0.01_NMTi_0.1_O are in excellent agreement (*R*_p_ < 1.95%, *R*_wp_ < 3.28%) (Figure 1f), and the detailed lattice parameters calculated by the Rietveld method are summarized in Tables S1 and S2 (Supporting Information). The apparent changes in lattice parameters indicate that K and Ti have been successfully doped into the host structure of NaNMO. According to previous studies, K^+^ and Ti^4+^ are most likely to occupy Na sites and Mn sites in the Na‐based layered oxide structure, respectively.^[^ ^11^, ^18^ ^]^ Figure 1g shows the lattice structures of NaNMO and NaK_0.01_NMTi_0.1_O constructed based on the refined lattice parameters. The difference between the layer spacing of NaK_0.01_NMTi_0.1_O (*d*_Na_ = 3.2020 Å, *d*_TM_ = 2.1513 Å) and NaNMO (*d*_Na_ = 3.1921 Å, *d*_TM_ = 2.1327 Å) can be attributed to the larger ionic radii of K^+^ (1.38 Å) and Ti^4+^ (0.60 Å) than those of Na^+^ (1.02 Å) and Mn^4+^ (0.53 Å), respectively.^[^ ^15^, ^17^ ^]^

### 2.2 Electrochemical Performances

**Figure 2:**
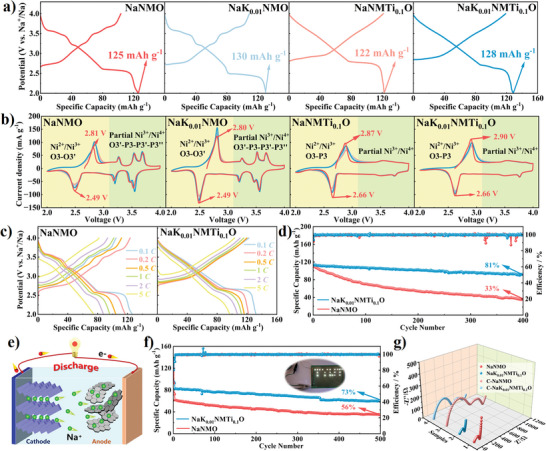
a) Galvanostatic charge/discharge curves (GCDs) of four materials in the first cycle. GCDs were obtained at 0.1 C. b) Cyclic voltammograms curves (CVs) of four materials in the first three cycles. c) GCDs of NaNMO and NaK_0.01_NMTi_0.1_O cycled at 0.1, 0.2, 0.5, 1.0, 2.0, and 5.0 C. d) Cycling performance of NaNMO and NaK_0.01_NMTi_0.1_O at 0.5 C in the voltage range of 2.0–4.0 V. e) Schematic diagram showing the discharge process and f) Cycling performance of NaNMO//HC and NaK_0.01_NMTi_0.1_O//HC full cells between 1.5 and 4.0 V during 500 cycles at 1 C. g) Nyquist plots of EIS and corresponding fitting curves of NaNMO and NaK_0.01_NMTi_0.1_O before and after cycling.

The electrochemical properties of all samples were investigated in Na^+^ half cell with a window of 2.0–4.0 V. NaK_0.01_NMO delivers a higher reversible capacity (130 vs 125 mAh g^−1^) than NaNMO (**Figure** **2**a), which is mainly due to that the large size of K^+^ pinned in the Na layer can reduce the strength of the Na‒O bond and the formation energy of the Na^+^ vacancy.^[^ ^15c^ ^]^ Although the introduction of inactive tetravalent Ti^4+^ (Ti^4+^: *t*_2g_^0^ *e*_g_^0^:*d* ^0^) will inevitably reduce the initial discharge capacity, the irreversible phase transition and Na^+^/vacancy ordering above 3 V are significantly suppressed due to the solid‐solution reaction of a large region.^[^ ^19^ ^]^ As shown in Figure 2b, four redox peaks (2.81/2.49, 3.28/3.18, 3.55/3.46, and 3.70/3.52 V) are observed in the CV of NaNMO, corresponding exactly to the four voltage plateaus in the charge/discharge curves. These four redox peaks are related to Ni^2+^/Ni^3+^/Ni^4+^ redox reaction, Na^+^/vacancy ordering, and O3‐O3′‐P3‐P3′‐P3″ multiple phase transition. During the cycle, the positions of the four pairs of redox peaks shifted significantly, indicating the poor reversibility of the multiphase transitions. When K^+^ is introduced into NaNMO, the intensity of the redox peak in the low‐voltage region (2.80/2.49 V) increases significantly, which demonstrates that K^+^ can promote Na^+^ diffusion during charging/discharging at lower voltages. With the introduction of Ti^4+^, the redox peaks of the CV curves above 3 V are significantly suppressed, and more symmetrical anodic/cathodic peaks at higher voltages (2.87/2.66 V) can be observed. Since the bond energy of Ti‒O bonds (666.5 KJ mol^−1^) is significantly higher than the Mn‒O (362 KJ mol^−1^) and Ni‒O bonds (391.6 KJ mol^−1^), sharing oxygen with Ti can stabilize the crystal structure and inhibit the multiphase transition due to the slip of the transition metal layers.^[^ ^20^ ^]^ Moreover, the smaller difference in ionic radii between Mn^4+^ and Ti^4+^ is favorable for inhibiting the transition metal layer ordering and multiphase transition.^[^ ^21^ ^]^

The discharge capacity of NaK_0.01_NMTi_0.1_O is much higher than that of NaNMO (97.1 vs 75.1 mAh g^−1^) at 5 C, which accounts for 73.6% and 59.4% of their initial capacity, respectively (Figure 2c; Figure S10, Supporting Information). Moreover, the energy density of NaK_0.01_NMTi_0.1_O is superior to NaNMO at various current densities due to the higher average voltage, e.g. 404.5 versus 375.1 Wh kg^−1^ at 0.1 C (Figure S11, Supporting Information). Although the initial discharge capacities of all samples exceed 120 mAh g^−1^ at 0.1 C, NaNMO experienced the fastest capacity decay, dropping to 37 mAh g^−1^ after 400 cycles, which corresponds to 33% of the initial capacity (Figure 2d). With the introduction of a single element (K or Ti), the capacity retention of NaK_0.01_NMO increases to 41%, while it reaches 62% for NaNMTi_0.1_O (Figure S12, Supporting Information). More importantly, the simultaneous introduction of both elements minimizes the initial capacity loss, and further shows a high discharge capacity of 128 mAh g^−1^ and excellent electrochemical reversibility for 1000 cycles (Figures S13, S14, and S15, Supporting Information). Based on the electrochemical properties of the different doping contents demonstrated in Figure S16, the optimized K and Ti contents are determined as 0.01 and 0.1, respectively.

In addition to the above remarkable electrochemical performances in sodium half cell, NaK_0.01_NMTi_0.1_O also exhibits excellent full cell performances, which initially demonstrates the practical commercial viability of the modified cathode (Figure 2e). The anode is pre‐cycled three times before assembling the full cell due to the low coulombic efficiency of hard carbon (HC) in the first cycle.^[^ ^22^ ^]^ Figures S17 and S18 (Supporting Information) show the rate performance and GCDs profiles of the HC anode, respectively. NaK_0.01_NMTi_0.1_O delivers a high reversible capacity of 95.5 mAh g^−1^ and a high average working voltage of ≈3.24 V, and can successfully illuminate commercial light‐emitting diodes (Figure 2f). Furthermore, the full cell with NaK_0.01_NMTi_0.1_O cathode shows better cyclic stability (retained capacity of 73% vs 56% after 500 cycles, at 1 C) than the reference NaNMO full cell. The value of the Warburg impedance *Z*_w_ depends on the interfacial impedance *R*_f_, the Na^+^ diffusion impedance in the electrolyte *R*_s_, and the charge transfer impedance *R*_ct_, etc.^[^ ^23^ ^]^ Electrochemical impedance spectroscopy (EIS) measurements were carried out to further assess the resistance of NaNMO and NaK_0.01_NMTi_0.1_O before and after cycling, and the detailed resistance data are shown in Table S3 (Supporting Information). The samples cycled for 50 cycles are labeled as C‐NaNMO and C‐NaK_0.01_NMTi_0.1_O, respectively. Since residual alkaline substances and by‐products on the particle surface may severely impair the reaction kinetics of the cathode, the *R*_f_ and *R*_ct_ of NaNMO and C‐NaNMO are significantly increased compared to the optimized materials, which confirms the enhanced electrode reaction kinetics for NaK_0.01_NMTi_0.1_O owing to its stronger structure and more stable interface (Figure 2g).^[^ ^8c^ ^]^

### 2.3 Structural Evolution

**Figure 3:**
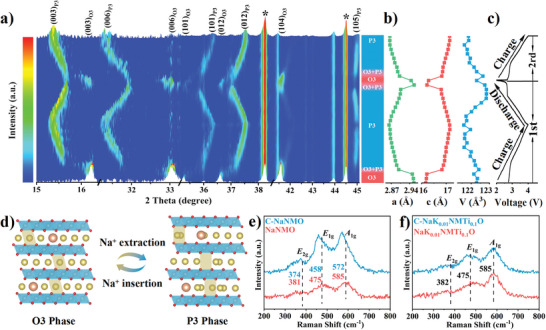
a) In situ XRD patterns of NaK_0.01_NMTi_0.1_O during the charge–discharge cycle. b) Calculated lattice parameters (*a*, *c*, and *V*) of NaK_0.01_NMTi_0.1_O at different voltages. c) Corresponding voltage profile. d) Schematic illustration showing the crystal structural evolution of NaK_0.01_NMTi_0.1_O upon Na^+^ extraction/insertion. Ex situ Raman patterns of e) NaNMO and f) NaK_0.01_NMTi_0.1_O.

In situ XRD experiments were carried out to investigate the effect of K/Ti co‐doping on structural evolution during Na^+^ extraction/insertion. As a classical O3‐type cathode, NaNi_0.5_Mn_0.5_O_2_ undergoes multiple phase transitions of O3‐O3′‐P3‐P3′‐P3″ during the cycle, which is consistent with the reported literature (Figure S22, Supporting Information).^[^ ^6^, ^24^ ^]^ For NaK_0.01_NMTi_0.1_O, upon initial charging, the (003), (006), (101), and (012) peaks of the O3 phase split into two whereas the intensity of the (104)_O3_ peak clearly decreases, revealing the formation of hexagonal P3 phase via the O3‐P3 biphasic reaction, corresponding to the plateau region at ≈2.8 V in GCD curve (**Figure** **3**a).^[^ ^25^ ^]^ Note that with the further extraction of Na^+^, the (104)_O3_ peak disappears, accompanied by the increased intensity of the (105)_P3_ peak, implying the complete disappearance of the O3 phase and the generation of a pure P3 phase. No new peaks appeared until charging to 4.0 V, indicating that the doping of K/Ti promotes the solid solution reaction (Figures S23 and S24, Supporting Information).^[^ ^26^ ^]^ It has been demonstrated that the phase transition is closely related to the local structure of the Na^+^.^[^ ^27^ ^]^ As Na^+^ is extracted from the lattice, the formation energy of Na^+^ in the prismatic sites is reduced, further causing the formation of the P3 phase.^[^ ^17^ ^]^ During the discharge, the XRD pattern undergoes an opposite evolution, demonstrating that high‐reversible structural evolution occurred during cycling. This result indicates the strong K‒O bond and Ti‒O bond can effectively mitigate the multiple phase transitions detrimental to long‐term stability. The calculated lattice parameters of NaK_0.01_NMTi_0.1_O at different charging/discharging states are further extracted from the in situ XRD. Figure 3b shows that the deviations of *a*, *c*, and *V* are 3.17%, 6.05%, and 0.59%, respectively. The smaller volume strain stabilizes the crystalline structure of the cathode, preventing particle damage and producing less inactive insulating material.^[^ ^28^ ^]^ Figure 3d briefly illustrates the structural evolution of NaK_0.01_NMTi_0.1_O during the intercalation/ deintercalation process.

Raman is an effective tool for revealing structural changes in electrodes and is widely used in the electrochemical analysis of battery systems.^[^ ^29^ ^]^ As shown in Figure 3e,f, the Raman peak centered at 475 and 585 cm^−1^, one of the typical features of layered cathode materials, represents the *E*_1g_ (O‒M‒O bending) and *A*_1g_ (M‒O stretching) modes, respectively.^[^ ^30^ ^]^ Moreover, the Raman peak located at 382 cm^−1^ corresponds to the *E*_2g_ of Na‒O vibrations.^[^ ^31^ ^]^ More importantly, the Raman peak of C‐NaNMO shifts to a lower wave number, which is possibly due to the lattice extension on the *c*‐axis and the change in the interatomic distance.^[^ ^32^ ^]^ In contrast, the Raman characteristic peak of C‐NaK_0.01_NMTi_0.1_O remains in its original position, implying excellent structural reversibility and further accounting for the remarkable cycling stability.

### 2.4 Interface Composition and Analysis

**Figure 4:**
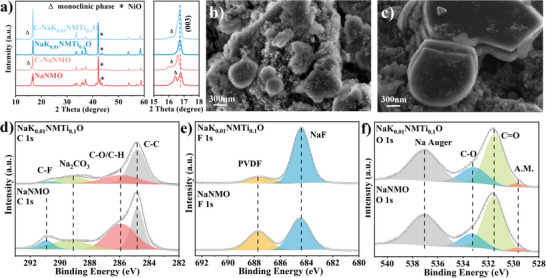
a) XRD diffraction patterns of NaNMO, NaK_0.01_NMTi_0.1_O, C‐NaNMO, and C‐NaK_0.01_NMTi_0.1_O. SEM images of b) C‐NaNMO and c) C‐NaK_0.01_NMTi_0.1_O. Comparison of the d) C 1*s*, e) F 1*s*, and f) O 1*s* XPS spectra of C‐NaNMO and C‐NaK_0.01_NMTi_0.1_O.

The electrode sheets after cycling were tested to further investigate the modification mechanism. The higher monoclinic phase peak in the XRD pattern of C‐NaNMO than that of C‐NaK_0.01_NMTi_0.1_ indicates the poor reversibility of structural evolution in C‐NaNMO (**Figure** **4**a). The cathode‐electrolyte interphase (CEI) is a significant constraint to improving the electrochemical performance of the cathode. We performed SEM and XPS tests to observe the CEI evolution of NaNMO and NaK_0.01_NMTi_0.1_O during cycling. The CEI layer generally consists of various products from the oxidative decomposition of electrolytes, dissolution of transition metals, and other side reactions.^[^ ^14b^ ^]^ As shown in Figure 4b,c, the particle surface and the interstices between the primary particles of C‐NaNMO are covered with a large amount of by‐products, which is due to the reconstruction of the surface crystal structure caused by the dissolution of the transition metal from the crystal lattice.^[^ ^14b^ ^]^ In contrast, for C‐NaK_0.01_NMTi_0.1_O, the surface remains relatively smooth and the morphology remains well maintained, indicating that the interface side reactions were effectively depressed.^[^ ^33^ ^]^ The XPS spectra of both electrodes show typical peaks associated with species such as PVDF, NaF, C‒O, C═O, Na_2_CO_3_, active material (A.M.), and amorphous carbon black. It has been demonstrated that the concentration of carbonate deposited on the cathode surface is proportional to the decomposition rate by the electrolyte consisting of carbonate functional groups.^[^ ^34^ ^]^ Hence, the low surface carbonate peaks of NaK_0.01_NMTi_0.1_O imply excellent interfacial stability (Figure 4d,f). It can be observed that the CEI layer of NaK_0.01_NMTi_0.1_O has a higher NaF content, which plays a major role in the overall interface stability. NaF‐rich CEI layer can facilitate Na^+^ diffusion and alleviate electrode cracking, due to the low Na^+^ diffusion energy barrier (0.12 eV) and high shear modulus (31.4 GPa) of NaF (Figure 4e; Figure S20, Supporting Information).^[^ ^35^ ^]^ The result demonstrates that the reduced surface oxygen peak and the larger C/O ratio of C‐NaNMO imply a thicker CEI layer formed on the cathode surface (Figure S21, Supporting Information).^[^ ^36^ ^]^ In short, proper K/Ti doping can induce the formation of a NaF‐rich, thin but stable CEI layer, thus conferring better cycling performance to NaK_0.01_NMTi_0.1_O.

### 2.5 Theoretical Calculation of K/Ti Co‐Doped Oxide Compounds

**Figure 5:**
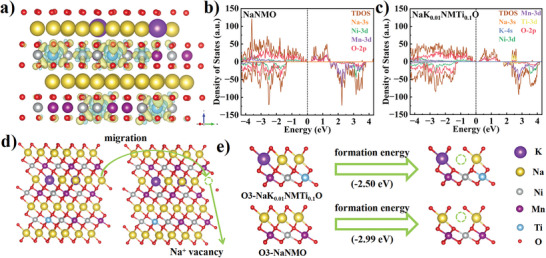
a) The charge density difference map of the Na layer and transition metal layer in NaK_0.01_NMTi_0.1_O structure. DOS of b) NaNMO and c) NaK_0.01_NMTi_0.1_O. d) Schematic that illustrates NaK_0.01_NMTi_0.1_O crystal structure with and without Na^+^ vacancy. e) The Na^+^ vacancy formation energy of NaNMO and NaK_0.01_NMTi_0.1_O.

To further understand the origin of improved air and structural stability of NaK_0.01_NMTi_0.1_O, DFT calculations were carried out to investigate the effect of K/Ti co‐doping on the intrinsic electronic structure and Na^+^ vacancy formation energy. The charge density difference map between the TM layer and the Na layer of NaK_0.01_NMTi_0.1_O structure is shown in **Figure** **5**a. The introduction of Ti^4+^ can effectively increase the number of electrons around oxygen and decrease the number of electrons around TM via promoting electronic delocalization (yellow represents the region of electron accumulation, and blue represents the region of electron reduction). The electron‐deficient state of the TM^n+^ can inhibit the spontaneous oxidation reactions and the release of lattice Na upon exposure to air.^[^ ^23^ ^]^ To understand the redox mechanism theoretically, DFT calculations were performed to obtain the Density of States (DOS) for the four materials. As shown in Figure 5b,c, and Figure S25 (Supporting Information), Ni 3*d* and O 2*p* orbitals dominate the electronic states near the Fermi level, suggesting that the electron transfer can be compensated by the redox reaction of Ni ions.^[^ ^28^ ^]^ Furthermore, the conduction band energy levels of the TM 3*d* orbitals and O 2*p* orbitals of NaK_0.01_NMTi_0.1_O are clearly shifted to higher energy levels, which implies that the energy level of the doped material is higher when gaining electrons during the discharge process (conduction band energy levels get electrons), that is, the discharge voltage is higher, in agreement with the above discussion (Figure S26, Supporting Information).^[^ ^37^ ^]^

The energy required to be absorbed when the intact crystal structure transforms into a structure containing Na^+^ vacancy is known as the Na^+^ vacancy formation energy. A lower Na^+^ vacancy formation energy means that less energy needs to be absorbed for Na^+^ extraction during charging. Therefore, we calculated and compared the Na^+^ vacancy formation energy of four materials to better understand the influence of doping elements on Na^+^ extraction. According to Figure S27 (Supporting Information), the Na^+^ vacancy formation energy around K^+^ (2.44 eV) of NaK_0.01_NMO is much lower than that of the pristine (2.99 eV), implying that K^+^ can enhance the diffusion of Na^+^ in the bulk phase via lowering the Na^+^ diffusion energy barrier in the low voltage region. The apparent decrease in Na^+^ vacancy formation energy may be due to the strong K‒O bond and the more open Na layer spacing.^[^ ^15c^ ^]^ It is also observed that the Na^+^ vacancy formation energy of NaK_0.01_NMTi_0.1_O (2.50 eV), although slightly higher than that of NaK_0.01_NMO, is still significantly lower than that of the pristine (Figure 5d,e). Namely, the co‐substituted NaK_0.01_NMTi_0.1_O provides a faster Na^+^ diffusion channel than NaNMO, delivering favorable electrochemical performance in the voltage region of 2.0–4.0 V.

## 3 Conclusion

In summary, we have proposed a K/Ti co‐substitution strategy to stabilize the O3‐type Na_0.99_K_0.01_Ni_0.5_Mn_0.4_Ti_0.1_O_2_ cathode for high‐energy and long‐cycle NIBs. The obtained NaK_0.01_NMTi_0.1_O shows a highly reversible O3‐P3 phase transition during charge‐discharge, and has an ultra‐low volume change (0.59%) upon charging to 4.0 V. Such enhancement is because that Ti^4+^ can inhibit the spontaneous oxidation reactions and the release of lattice Na via promoting electron delocalization. The lower Na^+^ vacancy formation energy (2.50 vs 2.99 eV) in NaK_0.01_NMTi_0.1_O than that in NaNMO facilitates the bulk Na^+^ diffusion. Hence, the modified NaK_0.01_NMTi_0.1_O delivers impressive electrochemical properties including high reversible capacity (128 mAh g^−1^), outstanding capacity retention (81% after 400 cycles for half cell, 73% after 500 cycles for full cell), and rate capability (97 mAh g^−1^ at 5 C). This multi‐ion synergistic modification strategy compensates for the single effect and side effects associated with single‐element doping and will open a new way to design advanced energy materials for electrochemical energy storage.

## 4 Experimental Section

### Cathode Synthesis

The Na_1‐_ *_x_* K*_x_* Ni_0.5_Mn_0.5‐_ *_y_* Ti*_y_* O_2_ (*x* = 0, 0.01; *y* = 0, 0.1) materials were synthesized by sol–gel method and labeled as NaNMO, NaK_0.01_NMO, NaNMTi_0.1_O, NaK_0.01_NMTi_0.1_O, respectively. A stoichiometric mixture of sodium acetate anhydrous (C_2_H_3_O_2_Na, Aladdin, AR, 99.0%), potassium acetate (C_2_H_3_KO_2_, Aladdin, AR, 99.0%), nickel acetate tetrahydrate (C_4_H_6_NiO_4_·4H_2_O, Aladdin, AR, 99.0%), manganese acetate tetrahydrate (C_4_H_6_MnO_4_·4H_2_O, Aladdin, AR, 99.0%), and citric acid (C_6_H_8_O_7_, Macklin, AR, 99.5%) were dissolved in the distilled water and stirred well to form metal salt solution and citric acid solution, respectively (M: CA = 1:1, M = Na + Ni + Mn + K + Ti). Subsequently, stoichiometric amounts of tetra butyl titanate (C_16_H_36_O_4_Ti, Aladdin, AR, ≥99.0%) and the metal salt solution were slowly added to the stirring citric acid solution. Then, the ammonia solution was dropped to form a mixed solution with pH = 8, and the obtained solution was continuously stirred and heated at 80 °C to form the gel. Finally, the gel was dried at 80 °C to form precursors which were sintered at a high temperature to obtain cathode materials. The specific calcination was as follows: the precursors were calcined at 500 °C for 5 h in the air to remove the citric acid in precursors; calcined at 900 °C for 12 h in the air; cooled to 700 °C at a rate of 1 °C min^−1^, and then quenched at room temperature. Additionally, to obtain air‐exposed materials, NaNMO and NaK_0.01_NMTi_0.1_O were exposed under the condition with a temperature of 25 °C and a humidity of 55% for 1 day or 2 days. The materials after 50 cycles were labeled as C‐NaNMO and C‐NaK_0.01_NMTi_0.1_O, respectively.

### Cathode Characterizations

To characterize the crystal structure of the synthesized materials, all the samples' X‐ray diffraction (XRD) patterns were observed on a Rigaku Ultima IV diffractometer with Cu K*α* radiation with the 2*θ* range of 10–80°. GSASII software was used to further Rietveld refinement of the XRD data as a means to investigate the lattice structure. The ex situ XRD test was performed on the electrodes at different voltages of charge/discharge obtained by dissembling the coin‐type cell in an argon‐filled glovebox. The in situ XRD analysis was performed using a homemade in situ cell equipped with thin aluminum windows. Scanning electron microscopy (SEM, JEOL JSM‐7001F) and high‐resolution transmission electron microscopy (HRTEM, JEOL, JEM‐2010F) were used to characterize the morphology and interlayer structure of the materials. The elemental distribution of the materials was identified by Energy‐dispersive X‐ray spectroscopy (EDS) mapping. To detect the electronic state of the elements on the surface, X‐ray photoelectron spectroscopy (XPS) data were gathered on an ESCALAB 250XI spectrometer. The surface functional groups and structural vibrational modes of the materials were characterized by Fourier Transform Infrared Spectrometer (FTIR, Thermo Scientific Nicolet iS20) and Raman (LabRam HR Evolution).

### Electrochemical Measurements

To prepare the working electrodes, the cathode material, Super P, and polyvinylidene fluoride (PVDF) were mixed well by grinding in a mass ratio of 8:1:1 and then dissolved in an appropriate amount of *N*‐methyl‐2‐pyrrolidone (NMP). The obtained slurry was uniformly applied to the Al foil and quickly dried on an electric heating plate at 60 °C. The obtained electrodes were further vacuum dried at 120 °C for 12 h and then punched into round slices with a diameter of 12 mm. The typical loading of active materials was 2.2 mg cm^−2^. To evaluate the electrochemical capability, the coin‐type (CR2025) half cell was assembled in an argon‐filled glovebox (MBraun, Germany, H_2_O and O_2_ < 0.5 ppm) with Na metal sheet as the anode, the synthetic electrodes as the cathode, glass fiber filter (Whatman) as the separator, and 1 m NaClO_4_ dissolved in ethylene carbonate (EC) and propylene carbonate (PC) (1:1 in volume, 5% fluoroethylene carbonate (FEC)) as the electrolyte. The coin‐type full cell was assembled using NaK_0.01_NMTi_0.1_O as the cathode and hard carbon as the anode. The hard carbon (HC) electrodes were prepared in the way same as the above working electrode. The N/P value of the full cell was set to 1.05, and the specific capacity of the full cell was calculated based on the mass of the cathode active material. The Galvanostatic charge/discharge cycling tests were carried out on a LAND (Wuhan, China) test instrument at room temperature between 2.0 and 4.0 V (1 C = 240 mA g^−1^). Cyclic voltammetry (CV) measurements were examined at a scan rate of 0.1 mV s^−1^, and electrochemical impedance spectroscopy (EIS) tests were measured with a frequency range from 100000 to 0.01 Hz and an amplitude of 5 mV using an electrochemical workstation (CHI660D, Shanghai, Chenhua). For the Galvanostatic intermittent titration technique (GITT) measurements, the coin‐type cell was charged/discharged at 0.1 C for 4 min and then executed a relaxation for 30 min.

### Computational Methods

All polarized density functional theory (DFT) calculations were performed with the Vienna Ab initio Simulation Package (VASP) in 96‐atom supercell models of Na_24_Ni_12_Mn_12_O_48_, Na_23_KNi_12_Mn_12_O_48_, Na_24_Ni_12_Mn_10_Ti_2_O_48_, and Na_23_KNi_12_Mn_10_Ti_2_O_48_.^[^ ^38^ ^]^ The Perdew–Burke–Ernzerhof revised for solids functional (PBEsol) had been applied to the exchange‐correlation function, using the generalized gradient approximation form (GGA‐PBE).^[^ ^39^ ^]^ The projector augmented wave (PAW) method was applied with the plane‐wave energy cutoff of 520 eV.^[^ ^40^ ^]^ All the atomic coordinates and lattice parameters were fully relaxed by the conjugate gradient algorithm, where the convergence criteria of the energy and force acting on each atom were less than 10^−5^ eV and 0.05 eV Å^−1^, respectively. The Monkhorst‐Pack scheme 2 × 3 × 1 *k*‐point mesh was used for relaxation. Due to the strongly correlated 3*d*‐electrons, the Hubbard *U* values for Ni 3*d*, Mn 3*d*, and Ti 3*d* were 6.30, 4.95, and 2.5 eV, respectively.^[^ ^41^ ^]^

## Conflict of Interest

The authors declare no conflict of interest.

## Author Contributions

L.‐R.W. – Resources, Investigation, Methodology, Writing‐Original Draft, Data Curation. Y.‐H.Z. – Resources, Writing‐Original Draft, Data Curation. Z.W. – Data curation, Resources, Formal analysis. J.T. – Data curation, Methodology. H.W. – Data curation, Formal analysis. H.Z. – Data curation, Investigation. S.X. – Data curation, Resources. L.C. – Data curation, Resources. X.D. – Conceptualization, Validation, Writing‐Review & Editing, Supervision, Project administration, Funding Acquisition. D.Z. – Conceptualization, Validation, Writing‐Review & Editing, Supervision, Project administration, Funding Acquisition. H.G. – Data curation, Resources, Validation, Supervision. Y.Y. – Conceptualization, Validation, Writing‐Review & Editing, Supervision, Project administration. Z.Z. – Conceptualization, Validation, Writing‐Review & Editing, Supervision, Project administration.

## Supporting information

Supporting Information

## Data Availability

The data that support the findings of this study are available from the corresponding author upon reasonable request.
