# Supplementary material for: Stabilized O3‐Type Layered Sodium Oxides with Enhanced Rate Performance and Cycling Stability by Dual‐Site Ti4+/K+ Substitution
Source: Adv Sci (Weinh). 2023 Sep 26;10(32):2304067. doi: 10.1002/advs.202304067 (PMC10646236; doi:10.1002/advs.202304067)
Supplement: Supplementary file 1 — Supporting Information [file ADVS-10-2304067-s001.pdf]

ADVANCED SCIENCE
Open Access

Supporting Information

for *Adv. Sci.*, DOI 10.1002/advs.202304067

Stabilized O3-Type Layered Sodium Oxides with Enhanced Rate Performance and Cycling Stability by Dual-Site Ti^{4+}/K^{+} Substitution

Lin-Rong Wu, Yu-Han Zhang, Zhen Wu, Jinlv Tian, Haorui Wang, Haijun Zhao, Shoudong Xu, Liang Chen, Xiaochuan Duan, Ding Zhang*, Huijuan Guo, Ya You* and Zhi Zhu**

Supporting Information

Stabilized O3-type Layered Sodium Oxides with Enhanced Rate Performance and Cycling Stability by Dual-Site Ti^{4+}/K^{+} Substitution

*Lin-Rong Wu, Yu-Han Zhang, Zhen Wu, Jinlv Tian, Haorui Wang, Haijun Zhao, Shoudong Xu, Liang Chen, Xiaochuan Duan**, *Ding Zhang**, *Huijuan Guo, Ya You**, *Zhi Zhu**

Lin-Rong Wu, Jinlv Tian, Haorui Wang, Haijun Zhao, Shoudong Xu, Xiaochuan Duan, Ding Zhang

College of Chemical Engineering and Technology, Taiyuan University of Technology, 79 Yingze West Street, Taiyuan 030024, P. R. China.

Email: duanxiaochuan@tyut.edu.cn, zhangding@tyut.edu.cn

Yu-Han Zhang

Qingdao Industrial Energy Storage Research Institute, Qingdao Institute of Bioenergy and Bioprocess Technology, Chinese Academy of Sciences, Qingdao 266101, P. R. China.

Yu-Han Zhang

School of Future Technology, University of Chinese Academy of Sciences, Beijing 100049, P. R. China.

Zhen Wu

State Key Laboratory for Mechanical Behavior of Materials, Xi'an Jiaotong University, Xi'an 710049, P.R. China.

Liang Chen, Xiaochuan Duan

College of Chemistry, Taiyuan University of Technology, 79 Yingze West Street, Taiyuan 030024, P. R. China.

Email: duanxiaochuan@tyut.edu.cn

Ding Zhang, Huijuan Guo

School of Chemical Engineering and Pharmacy, Wuhan Institute of Technology, Wuhan 430205, P. R. China.

Email: zhangding@tyut.edu.cn

Ya You

State Key Laboratory of Advanced Technology for Materials Synthesis and Processing, Wuhan University of Technology, Wuhan 430070, P. R. China.

Email: youya@whut.edu.cn

Ya You

International School of Materials Science and Engineering, School of Materials and Microelectronics, Wuhan University of Technology, Wuhan 430070, P. R. China.

Email: youya@whut.edu.cn

Zhi Zhu

School of Energy and Environment, Southeast University, Nanjing 211189, P. R. China.

Email: zhizhu@seu.edu.cn

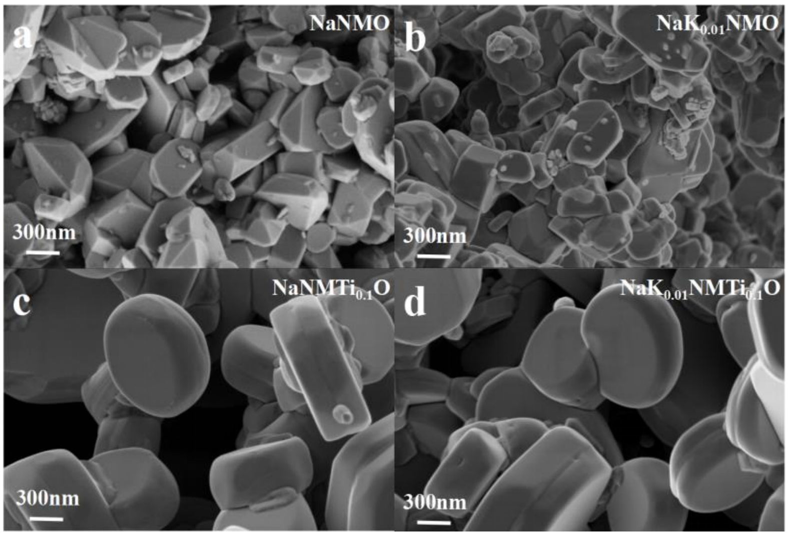

Figure S1. The SEM images of cathode materials of a) NaNMO, b) $NaK_{0.01}NMO$, c) $NaNMTi_{0.1}O$, and d) $NaK_{0.01}NMTi_{0.1}O$.

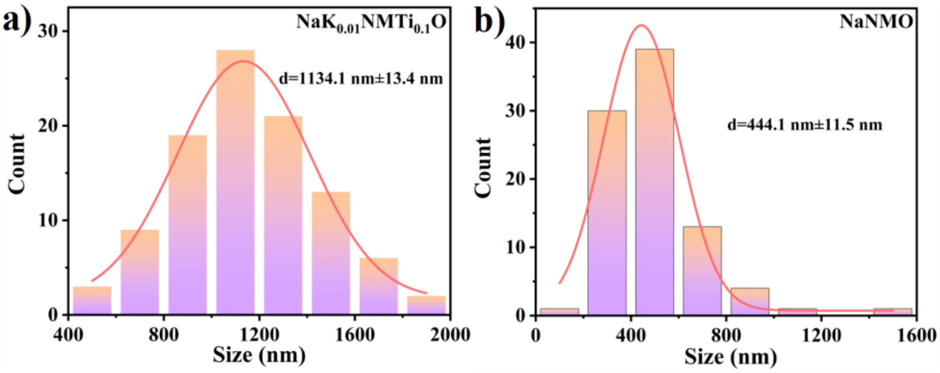

Figure S2. The particle size statistical distribution diagrams of a) NaNMO and b) $NaK_{0.01}NMTi_{0.1}O$.

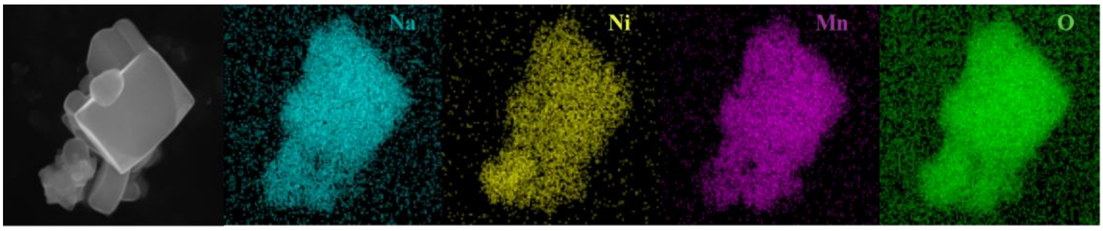

Figure S3. SEM image and corresponding EDS mappings of NaNMO.

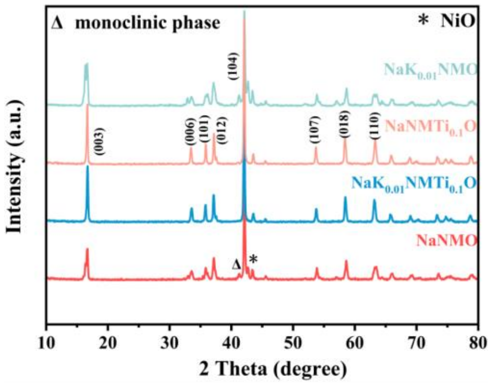

Figure S4. XRD diffraction patterns of NaNMO, $NaK_{0.01}NMO$, $NaNMTi_{0.1}O$, and $NaK_{0.01}NMTi_{0.1}O$.

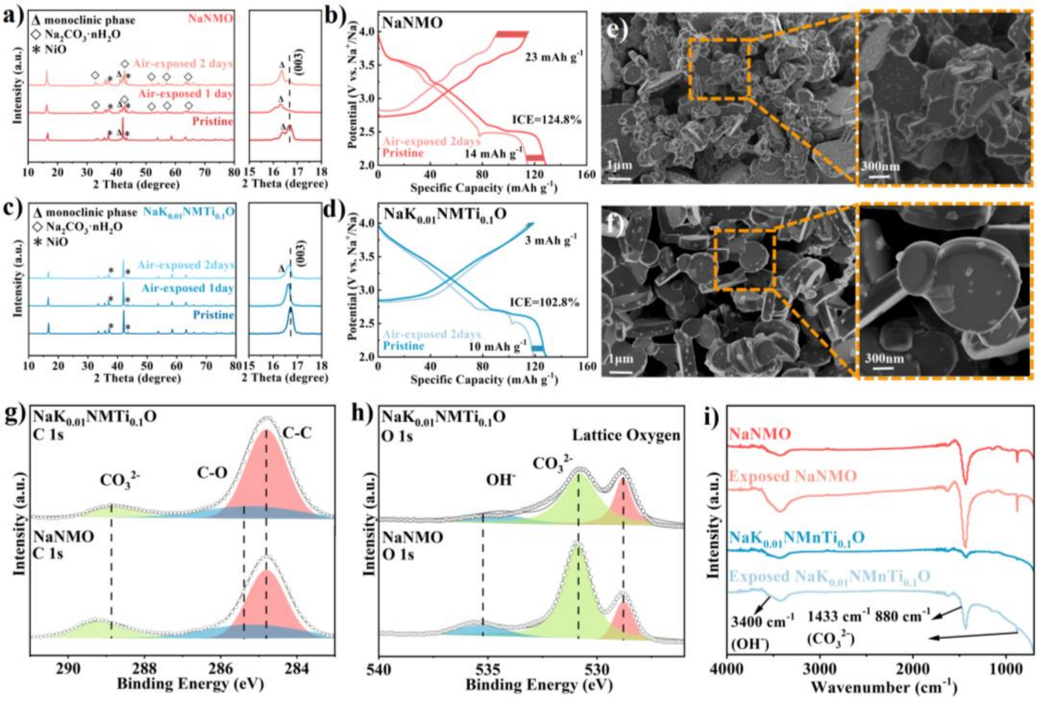

Figure S5. a) The XRD patterns of NaNMO and Air-exposed NaNMO and local magnified region of 15°-18° on the right. b) The first GCDs of NaNMO and Air-exposed NaNMO. c) The XRD patterns of $NaK_{0.01}NMTi_{0.1}O$ and Air-exposed $NaK_{0.01}NMTi_{0.1}O$ and local magnified region of 15°-18° on the right. d) The first GCDs of $NaK_{0.01}NMTi_{0.1}O$ and Air-exposed $NaK_{0.01}NMTi_{0.1}O$. SEM images of e) Air-exposed NaNMO and f) Air-exposed $NaK_{0.01}NMTi_{0.1}O$. XPS analysis of g) C 1*s* and h) O 1*s*. Where $NaK_{0.01}NMTi_{0.1}O$ is displayed at the top and NaNMO is at the bottom. i) FTIR spectra of NaNMO and $NaK_{0.01}NMTi_{0.1}O$ before and after the air exposure for 2 days.

1. Air stability analysis

To investigate the effect of K/Ti co-doping on the air stability of NaNMO, we performed a comprehensive characterization by XRD, SEM, and electrochemical properties to provide information on the structure-interface-property relationship between the fresh and air-exposed samples. As shown in Figure S5a, the XRD pattern of fresh NaNMO showed splitting of the (003) and (104) peaks, which indicates the generation of the monoclinic phase O3'-$Na_{1-x}NMO$ and surface residual bases. After

be exposed to air for 2 days, the diffraction peaks of the aged NaNMO become significantly broader and weaker, which demonstrates the reduced crystallinity. Further, with the appearance of the monoclinic phase, the O3 peak shifts to a lower angle due to the massive spontaneous escape of bulk sodium. The escaped sodium species tend to react with H_2O and CO_2 in air, and then produce large amounts of residual alkali impurities on the surface.[1] In contrast, the aged $NaK_{0.01}NMTi_{0.1}O$ maintains the original XRD pattern without any new peaks (Figure S5c), which indicates the successful suppression of the unfavorable bulk Na escape and H_2O embedding. The NaNMO provides an initial charge capacity of 115.1 mAh g^{-1}, which drops dramatically to 92.0 mAh g^{-1} after 2 days of air exposure (Figure S5b). The large capacity loss of up to 23.1 mAh g^{-1} implies that spontaneously active lattice sodium escape is significantly present in the air-exposed O3-type material. Note that, suffering from severe spontaneous escape of Na, the initial charging capacity of air-exposed NaNMO is much lower than the theoretical capacity, thus delivering an unusually high initial Coulomb efficiency of 124.8%. In contrast, the aged $NaK_{0.01}NMTi_{0.1}O$ has a higher initial charging capacity and thus shows an excellent initial coulombic efficiency (102.8%) (Figure S5d). Additionally, the morphological evolution of the air-exposed samples was further investigated via SEM. The aged NaNMO particles were heavily agglomerated and covered with many significant particulate substances on the surface (Figure S5e), which could be attributed to the generation of Na-resistive surface species during exposure to air.[2] Additional evidence of increased air stability was provided by the minimal number of alkaline impurities that were found on the surface of $NaK_{0.01}NMTi_{0.1}O$ (Figure S5f). The above results suggest that the K/Ti co-doping can effectively inhibit the chemical sensitivity of NaNMO to H_2O and CO_2, which results in more active Na^+ retained in the structure, less residual alkaline material generated on the surface, and excellent structural and cycling stability (Figure S6).

The specific composition of the surface products of the particles was examined using XPS to further evaluate the impact of K/Ti co-doping on the transition metal

valence of NaNMO. The peaks at 289.1, 285.5, and 284.8 eV in the C 1*s* spectra are attributed to carbonate, amorphous C–O, and C–C, respectively, while the peaks in the O 1*s* spectra are primarily generated from absorbed OH^-, carbonate, and lattice oxygen (Figures S5g, S5h, and S5i).[3] For these fresh samples, the minute amount of alkaline material on the surface may have resulted from inevitable residual Na_2CO_3 during transferring the material from the furnace to the sealed vial in air (< 1 min) or during natural cooling. It can be observed that the peak intensity of CO_3^{2-} in the $NaK_{0.01}NMTi_{0.1}O$ is much lower than that of the pristine. The characteristic peaks of FTIR around 1433 cm^{-1} and 880 cm^{-1} have been attributed to CO_3^{2-}. After 2 days of air exposure, the peak intensities of carbonates of NaNMO and $NaK_{0.01}NMTi_{0.1}O$ are more robust than those of fresh samples. However, the signal intensity of CO_3^{2-} or HCO_3^- species of $NaK_{0.01}NMTi_{0.1}O$ was significantly lower than that of NaNMO, demonstrating the air stability of $NaK_{0.01}NMTi_{0.1}O$ is better than that of pristine. Furthermore, the intensity variation of the peak at 3400 cm^{-1} has a similar trend to that of CO_3^{2-}, which was positively correlated with the degree of hydration of the cathode material.[4] Additionally, we detected K^+ and Ti^{4+}, which is in line with our predictions (Figures S7 and S8).

Based on the significant difference in the Fermi energy levels between Ni^{2+}/Mn^{4+} and Ti^{4+}, the introduction of Ti^{4+} can effectively increase the valence state of Ni via inhibiting charge localization (the electronic delocalization decreases the number of electrons around Ni).[1, 5] As shown in Figure S9, while the peaks of Mn 2*p* do not shift significantly, the binding energy of Ni 2*p* shifts to higher values, which verifies that the higher oxidation state of Ni in $NaK_{0.01}NMTi_{0.1}O$. This high-valence Ni ion facilitates the suppression of spontaneous oxidation reactions, resulting in superior air stability, as clarified in the DFT calculations.

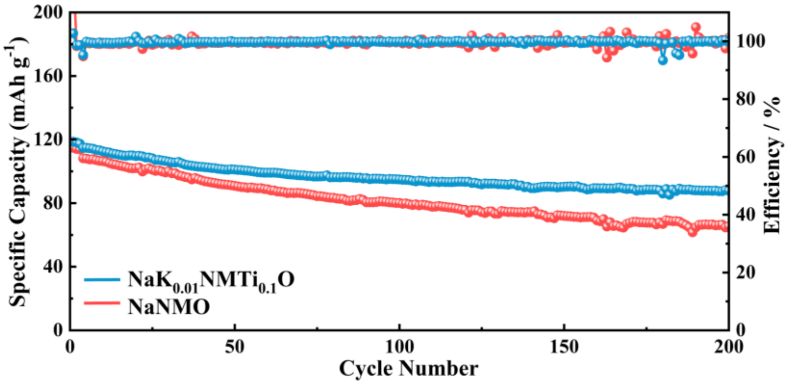

Figure S6. Cycling performance comparison of Air-exposed NaNMO and Air-exposed $NaK_{0.01}NMTi_{0.1}O$ during 200 cycles at 0.5 C in voltage range of 2.0-4.0 V.

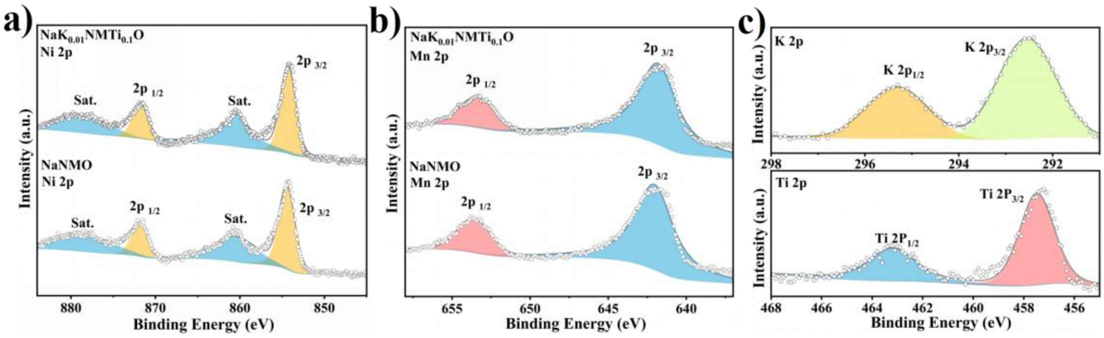

Figure S7. XPS analysis of a) Ni 2*p*, b) Mn 2*p*, and c) K 2*p* and Ti 2*p*. Where $NaK_{0.01}NMTi_{0.1}O$ is displayed at the top and NaNMO is at the bottom.

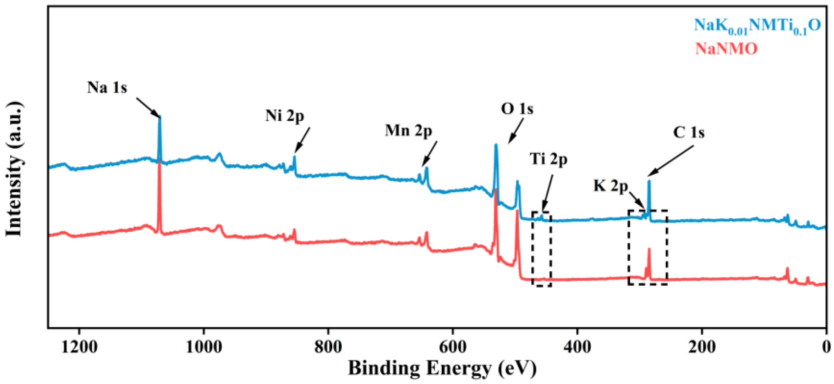

Figure S8. The XPS surveys of NaNMO and $NaK_{0.01}NMTi_{0.1}O$.

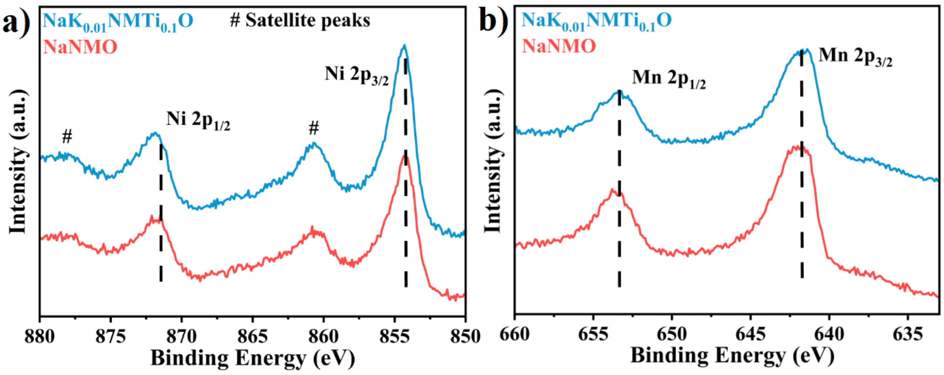

Figure S9. Comparison of the a) Ni 2*p* and b) Mn 2*p* XPS spectra of NaNMO and $NaK_{0.01}NMTi_{0.1}O$.

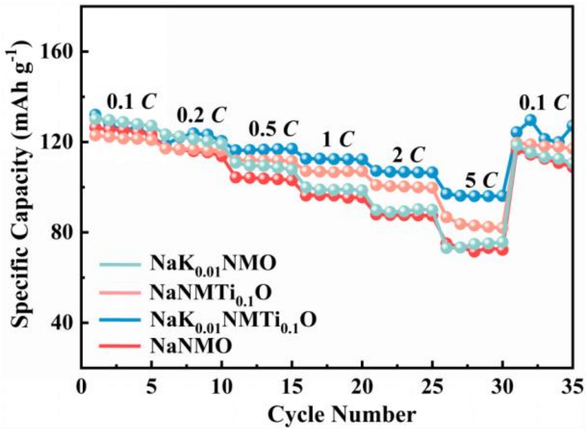

Figure S10. Rate performance comparison of NaNMO, $NaK_{0.01}NMO$, $NaNMTi_{0.1}O$, and $NaK_{0.01}NMTi_{0.1}O$ in voltage range of 2.0-4.0 V.

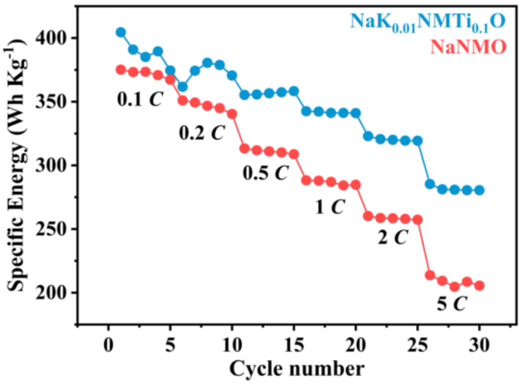

Figure S11. Specific energy comparison of NaNMO and $NaK_{0.01}NMTi_{0.1}O$ at different current densities in voltage range of 2.0-4.0 V.

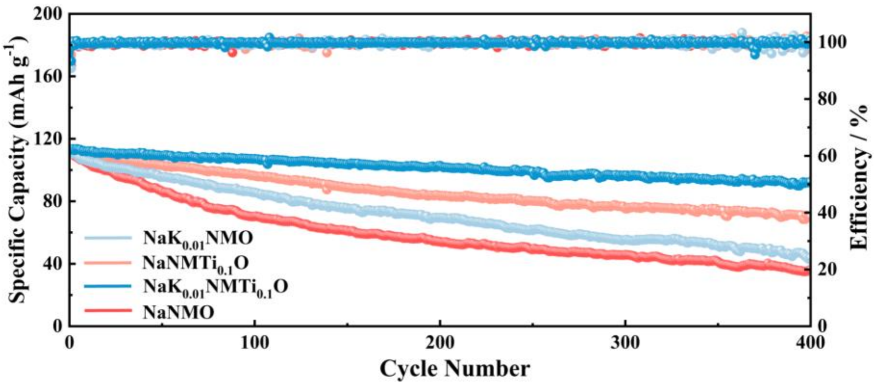

Figure S12. Cycling performance comparison of NaNMO, $NaK_{0.01}NMO$, $NaNMTi_{0.1}O$, and $NaK_{0.01}NMTi_{0.1}O$ during 400 cycles at 0.5 C between 2.0 V and 4.0 V.

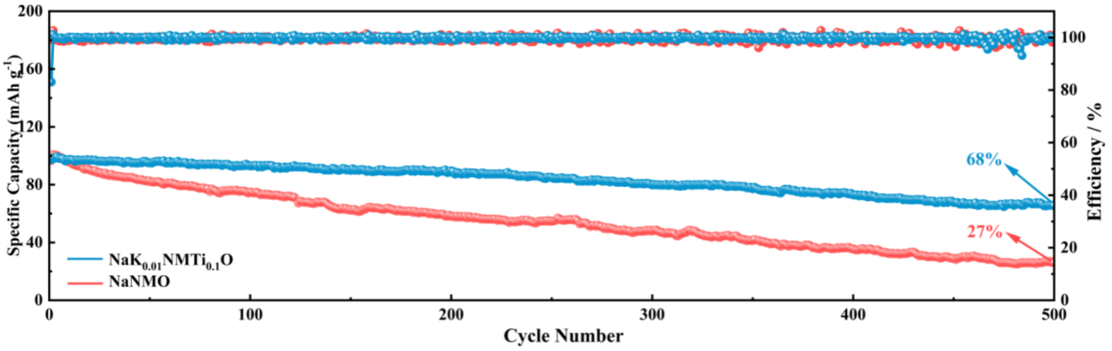

Figure S13. Cycling performance comparison of NaNMO and $NaK_{0.01}NMTi_{0.1}O$ during 500 cycles at 5 C in voltage range of 2.0-4.0 V.

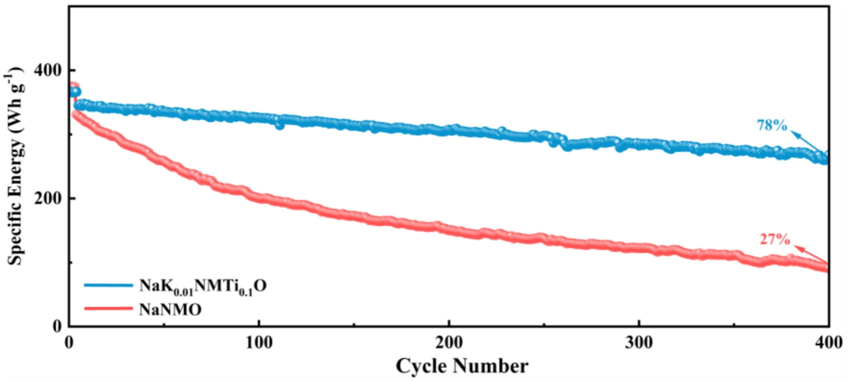

Figure S14. Specific energy comparison of NaNMO and $NaK_{0.01}NMTi_{0.1}O$ in voltage range of 2.0-4.0V during 400 cycles at 0.5 C.

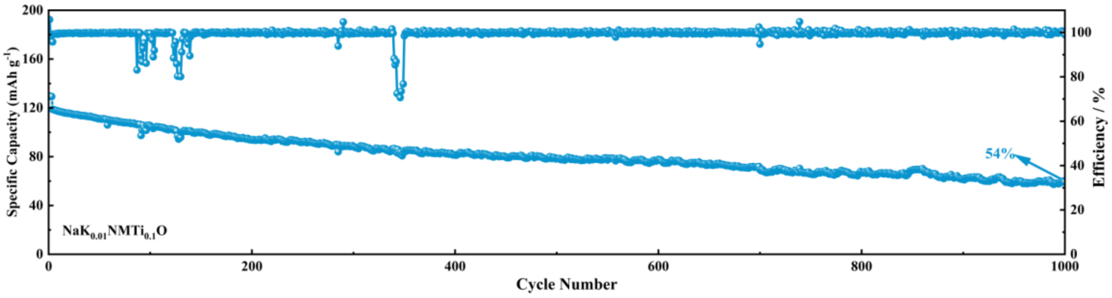

Figure S15. Cycling performance of $NaK_{0.01}NMTi_{0.1}O$ during 1000 cycles at 0.5 C in voltage range of 2.0-4.0 V.

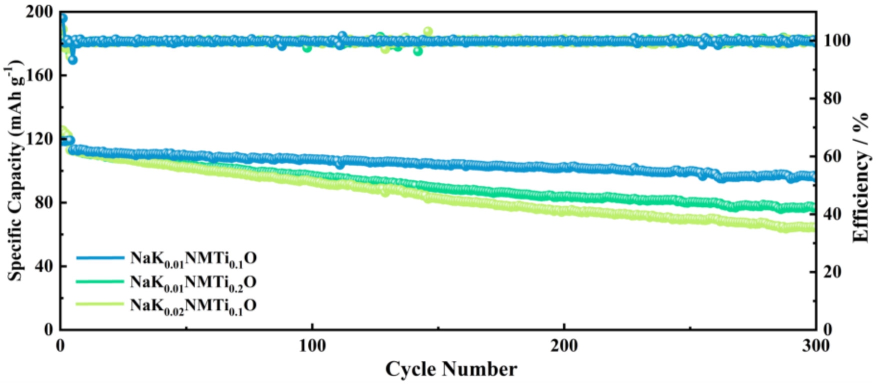

Figure S16. Cycling performance comparison of $NaK_{0.01}NMTi_{0.1}O$, $NaK_{0.02}NMTi_{0.1}O$, and $NaK_{0.01}NMTi_{0.2}O$ during 300 cycles at 0.5 C between 2.0 V and 4.0 V.

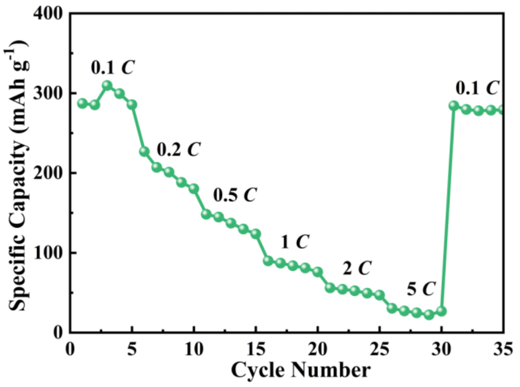

Figure S17. Rate performance of hard carbon.

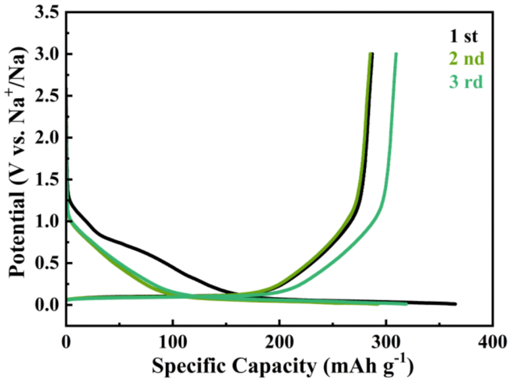

Figure S18. Galvanostatic charge/discharge curves (GCDs) of hard carbon.

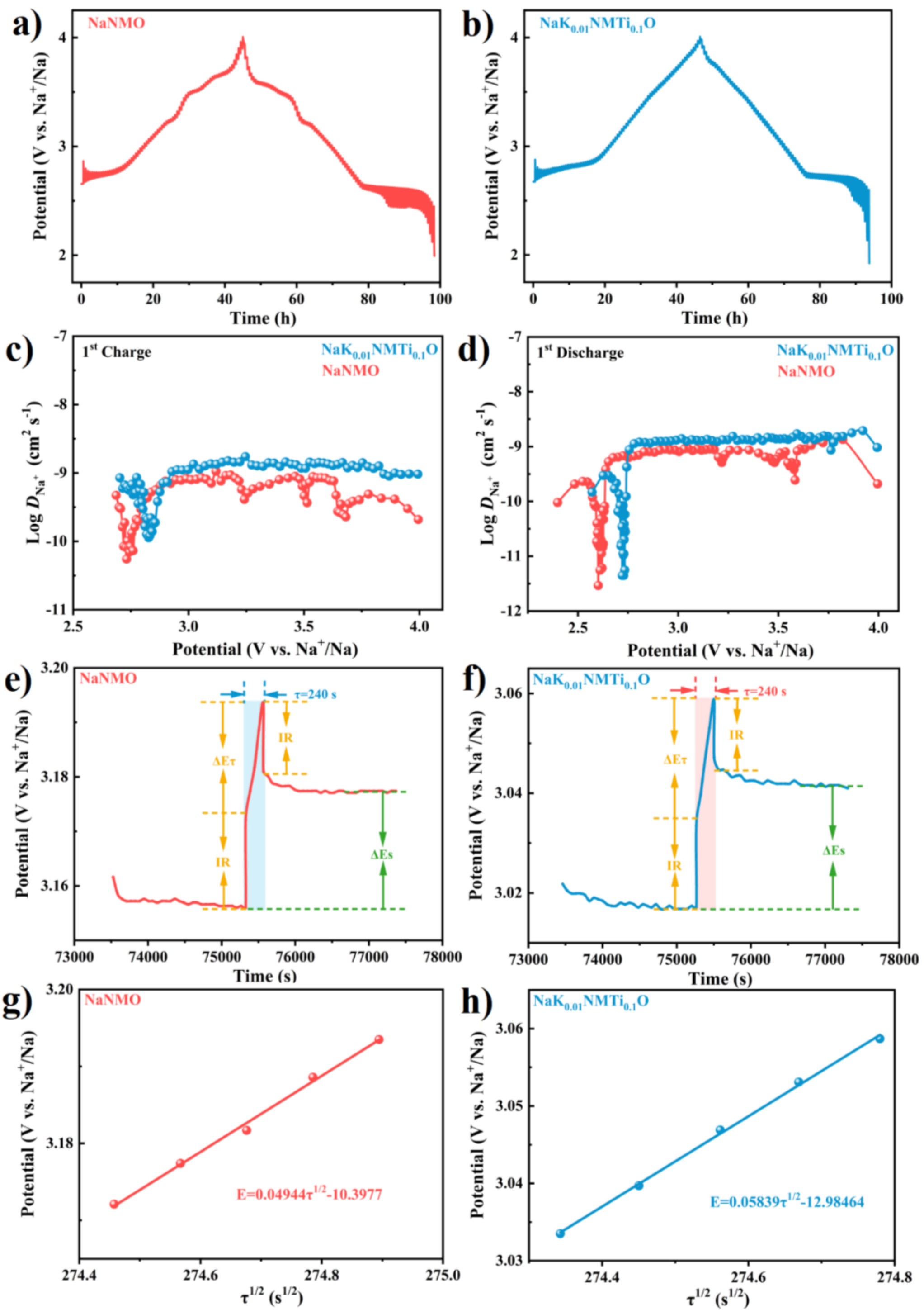

Figure S19. a, b) GITT profiles for the charge-discharge process of the initial cycles for NaNMO and $NaK_{0.01}NMO$, respectively. The Na^+ diffusion coefficient (D_{Na^+}) values are calculated for NaNMO and $NaK_{0.01}NMO$ at charge c) and discharge d) processes. Individual GITT titration curves of e) NaNMO and f) $NaK_{0.01}NMO$. Linear fit of $\tau^{1/2}$ and E during GITT titration of g) NaNMO and h) $NaK_{0.01}NMO$.

2. Kinetic analysis

To grasp more information about the kinetic processes, the diffusion behavior of Na^+ in NaNMO and $NaK_{0.01}NMTi_{0.1}O$ was analyzed by the GITT. The voltage change during relaxation on the GITT charge-discharge curve indicates the overpotential during the electrochemical reaction.[6] With reference to the GITT curves of both materials, the curve of $NaK_{0.01}NMTi_{0.1}O$ has a lower overpotential and therefore has faster kinetic properties.[6a] The calculated Na^+ diffusion coefficients D_{Na^+} in the two samples are compared in Figure S19, and it can be observed that the D_{Na^+} in $NaK_{0.01}NMTi_{0.1}O$ is significantly larger than the pristine throughout the cycling process, which well demonstrates the fast Na^+ diffusion and explains the enhanced rate performance of $NaK_{0.01}NMTi_{0.1}O$. Due to the O3-P3 biphasic reaction, a sharp drop in D_{Na^+} occurs between 2.5 and 3 V.[7]

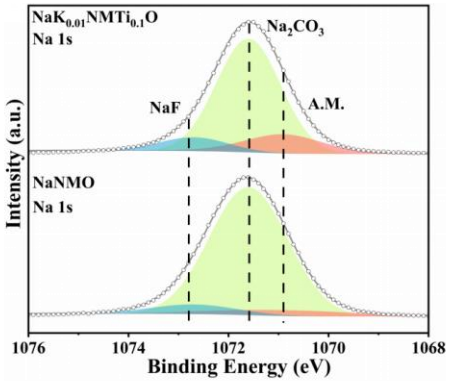

Figure S20. Comparison of the Na 1*s* XPS spectra of C-NaNMO and C-$NaK_{0.01}NMTi_{0.1}O$.

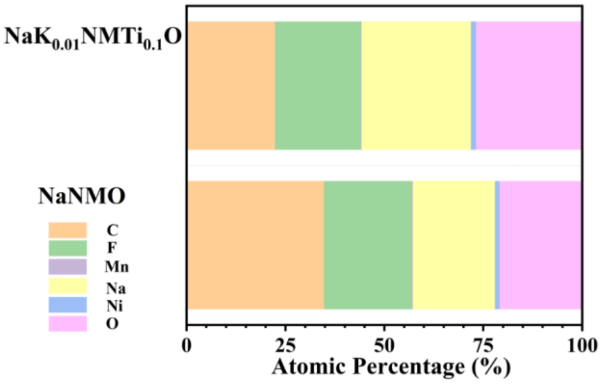

Figure S21. Atomic concentrations of the main elements comprising the CEI.

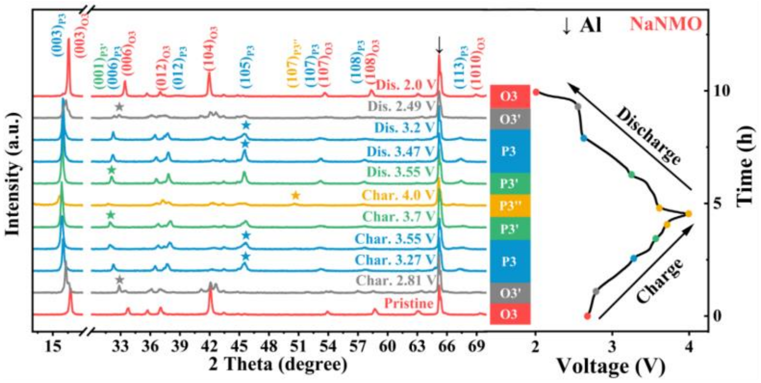

Figure S22. Ex-situ XRD patterns of NaNMO during the initial charge-discharge process at 0.1 C in the voltage range of 2.0-4.0 V.

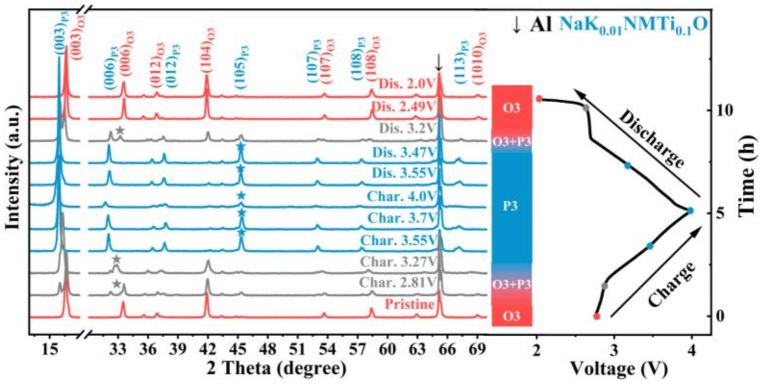

Figure S23. Ex-situ XRD patterns of $NaK_{0.01}NMTi_{0.1}O$ during the initial charge-discharge process at 0.1 C in the voltage range of 2.0-4.0 V.

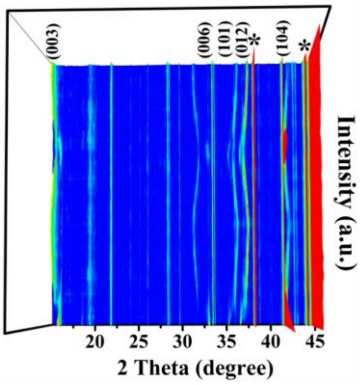

Figure S24. In-situ XRD patterns of $NaK_{0.01}NMTi_{0.1}O$ during charge-discharge cycle.

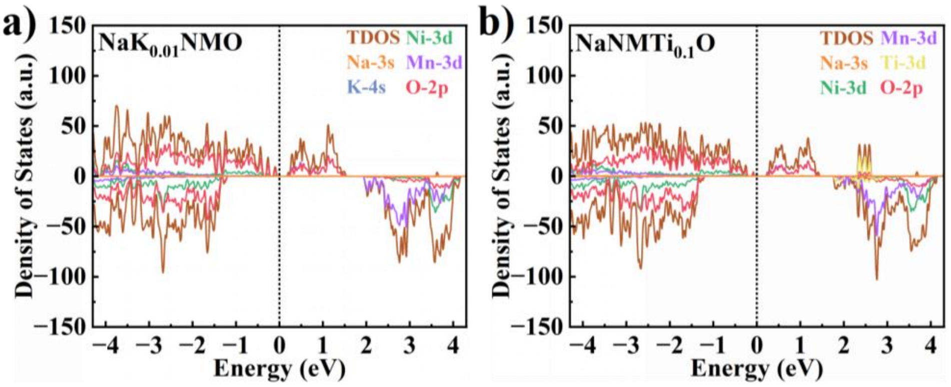

Figure S25. DOS of a) $NaK_{0.01}NMO$ and b) $NaNMTi_{0.1}O$.

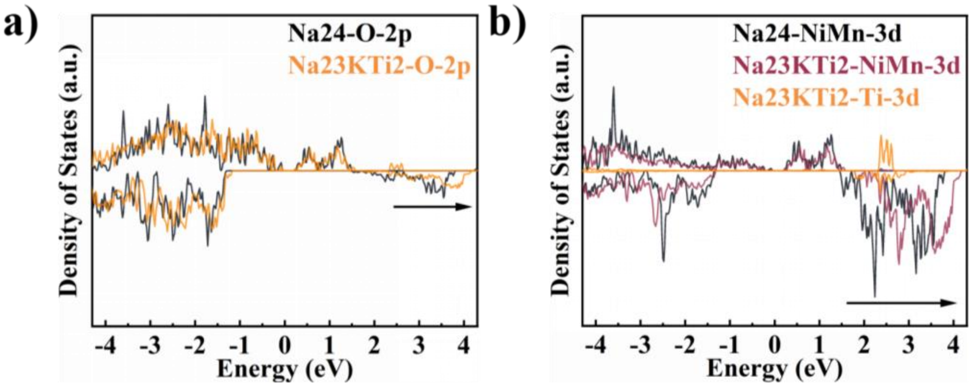

Figure S26. DOS comparison of a) O 2*p* and b) TM 3*d*.

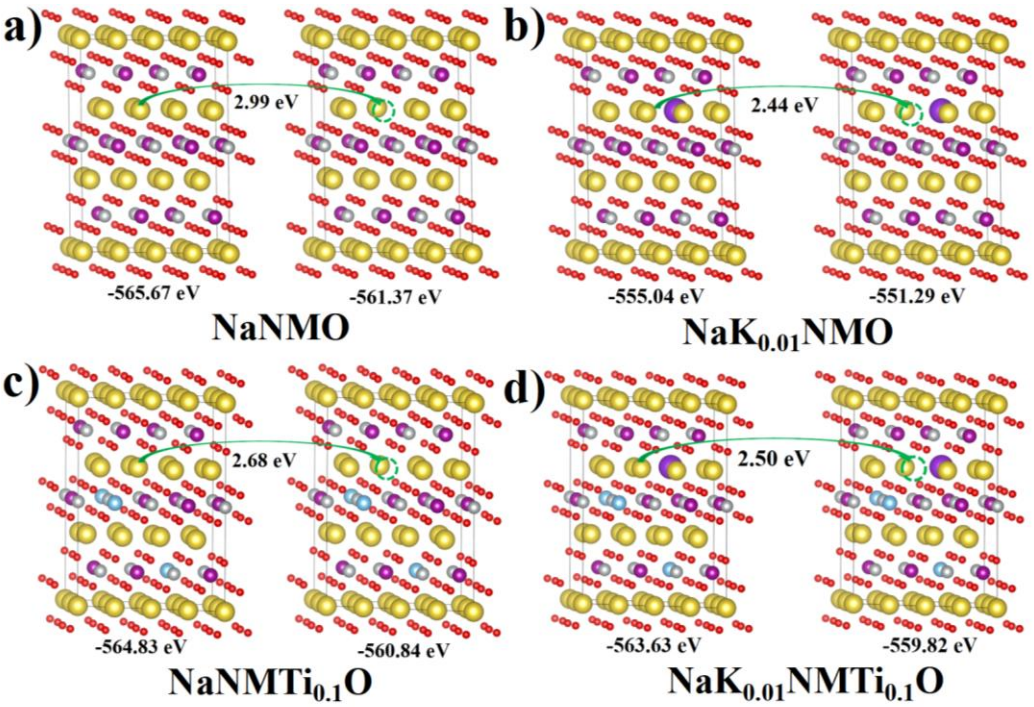

Figure S27. Schematic that illustrates the Na^+ vacancy formation energy of a) NaNMO, b) $NaK_{0.01}NMO$, c) $NaNMTi_{0.1}O$, and d) $NaK_{0.01}NMTi_{0.1}O$.

E(Na) = -1.31 eV

NaNMO: E1=E(Na23)+E(Na)-E(Na24)= -561.37+(-1.31)–(-565.67)= 2.99 eV

$NaK_{0.01}NMO$: E1=E(Na22K)+E(Na)-E(Na23K)= -551.29+(-1.31)–(-555.04)= 2.44 eV

$NaNMTi_{0.1}O$: E1=E(Na23)+E(Na)- E(Na24)= -560.84+(-1.31)–(-564.83)= 2.68 eV

$NaK_{0.01}NMTi_{0.1}O$: E1=E(Na22KNi12Mn10Ti2O48)+E(Na)-E(Na23KTi2)=-559.82+(-1.31)–(-563.63)= 2.50 eV

Table S1. Crystallographic parameters of $NaNi_{0.5}Mn_{0.5}O_2$ refined by the Rietveld method.

Atom	Site	x	y	z	Occupation
**Na**	3a	0	0	0	1.0
**Ni**	3b	0	0	1/2	0.5
**Mn**	3b	0	0	1/2	0.5
**O**	6c	0	0	0.233420	1.0
**a=b=2.94652 Å c=15.97425 Å V=120.107 Å^3 R_p=1.784 % R_{wp}=3.196 %**					

Table S2. Crystallographic parameters of $Na_{0.99}K_{0.01}Ni_{0.5}Mn_{0.4}Ti_{0.1}O_2$ refined by the Rietveld method.

Atom	Site	x	y	z	Occupation.
**Na**	3a	0	0	0	0.99
**K**	3a	0	0	0	0.01
**Ni**	3b	0	0	1/2	0.5
**Mn**	3b	0	0	1/2	0.4
**Ti**	3b	0	0	1/2	0.1
**O**	6c	0	0	0.233420	1.0
**a=b=2.95258 Å c=16.0607 Å V=121.254 Å^3 R_p=1.947 % R_{wp}=3.274 %**					

Table S3. Resistance of NaNMO, $NaK_{0.01}NMTi_{0.1}O$, C-NaNMO, and C-$NaK_{0.01}NMTi_{0.1}O$ cathodes obtained by fitting the Nyquist plots with an equivalent circuit.

Samples	R_s (Ω)	R_f (Ω)	R_{ct} (Ω)
**NaNMO**	4.189	31.86	81.07
**$NaK_{0.01}NMTi_{0.1}O$**	4.253	20.02	82.06
**C-NaNMO**	14.91	557.3	209.7
**C-$NaK_{0.01}NMTi_{0.1}O$**	5.078	425.7	107.8
**Circuit diagram for EIS fitting**	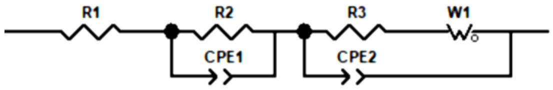		

R_s: Electrolyte impedance

R_f: Solid-state electrolyte interfacial impedance

R_{ct}: Charge transfer impedance

Table S4. Performance comparison of this work with previously reported the O3-$NaNi_{0.5}Mn_{0.5}O_2$ prepared by other doping modification methods.

**Doped elements**	**Sample**	**1st Discapacity Capacity (Current density, voltage range)**	**Capacity retention Cycle performance**	**Rate Performance**	**Reference Year**
K/Ti	$Na_{0.99}K_{0.01}Ni_{0.5}Mn_{0.4}Ti_{0.1}O_2$	128.8 mAh g^{-1} (24 mA g^{-1}, 2.0-4.0 V)	81.5 %, 120 mA g^{-1}, 400 cycles	97.1 mAh g^{-1} (1200 mA g^{-1})	This work
High entropy	$NaNi_{0.1}Mn_{0.15}Co_{0.2}Cu_{0.1}Fe_{0.1}Li_{0.1}Ti_{0.15}Sn_{0.1}O_2$	115 mAh g^{-1} (10 mA g^{-1}, 2.0-4.1 V)	82.7 %, 160 mA g^{-1}, 1000 cycles	93 mAh g^{-1} (160 mA g^{-1})	[8]2022
Sb	$NaNi_{0.5}Mn_{0.49}Sb_{0.01}O_2$	120 mAh g^{-1} (140 mA g^{-1}, 2.0-4.0 V)	96 %, 160 mA g^{-1}, 100 cycles	-	[9]2022
Fe/Mg	$NaNi_{0.35}Fe_{0.2}Mg_{0.05}Mn_{0.4}O_2$	129.4 mAh g^{-1} (24 mA g^{-1}, 2.0-4.0 V)	86 %, 240 mA g^{-1}, 150 cycles	73.7 mAh g^{-1} (1200 mA g^{-1})	[10]2021
Zn	$NaNi_{0.47}Zn_{0.03}Mn_{0.5}O_2$	113 mAh g^{-1} (120 mA g^{-1}, 2.0-4.0 V)	80 %, 120 mA g^{-1}, 150 cycles	82 mAh g^{-1} (1200 mA g^{-1})	[11]2021
Al	$NaAl_{0.02}(Ni_{0.5}Mn_{0.5})_{0.98}O_2$	130 mAh g^{-1} (24 mA g^{-1}, 2.0-4.0 V)	63.2 %, 240 mA g^{-1}, 200 cycles	90 mAh g^{-1} (480mA g^{-1})	[12]2020
Ti/Zr	$NaNi_{0.45}Mn_{0.3}Ti_{0.2}Zr_{0.05}O_2$	135.1 mAh g^{-1} (24 mA g^{-1}, 2.0-4.0 V)	70 %, 12 mA g^{-1}, 200 cycles	96 mAh g^{-1} (480 mA g^{-1})	[13]2020
Zn	$NaNi_{0.2}Fe_{0.35}Mn_{0.4}Zn_{0.05}O2$	146.6 mAh g^{-1} (12 mA g^{-1}, 1.5-4.2V)	63.9 %, 240 mA g^{-1}, 200 cycles	46 mAh g^{-1} (1200 mA g^{-1})	[14]2019
Li/Cu/Mg	$NaLi_{0.05}Ni_{0.3}Mn_{0.5}Cu_{0.1}Mg_{0.05}O_2$	≈ 130 mAh g^{-1} (62.5 mA g^{-1}, 2.0-4.0 V)	91.9 %, 625 mA g^{-1}, 600 cycles	71.8 mAh g^{-1} (6250 mA g^{-1})	[15]2018
Cu/Ti	$NaNi_{0.4}Cu_{0.1}Mn_{0.4}Ti_{0.1}O_2$	≈ 130 mAh g^{-1} (12 mA g^{-1}, 2.0-4.0V)	70.2 %, 240 mA g^{-1}, 500 cycles	81 mAh g^{-1} (2400 mA g^{-1})	[5b]2017
Ti	$NaNi_{0.5}Mn_{0.2}Ti_{0.3}O_2$	135 mAh g^{-1} (12 mA g^{-1}, 2.0-4.0V)	85 %, 240 mA g^{-1}, 200 cycles	≈ 95 mAh g^{-1} (1200 mA g^{-1})	[16]2017

Supplementary References

[1] H.-R. Yao, X.-G. Yuan, X.-D. Zhang, Y.-J. Guo, L. Zheng, H. Ye, Y.-X. Yin, J. Li, Y. Chen, Y. Huang, Z. Huang, Y.-G. Guo, *Energy Storage Mater.* **2023**, *54*, 661.

[2] X.-G. Yuan, Y.-J. Guo, L. Gan, X.-A. Yang, W.-H. He, X.-S. Zhang, Y.-X. Yin, S. Xin, H.-R. Yao, Z. Huang, Y.-G. Guo, *Adv. Funct. Mater.* **2022**, *32*, 2111466.

[3] F. Ding, Q. Meng, P. Yu, H. Wang, Y. Niu, Y. Li, Y. Yang, X. Rong, X. Liu, Y. Lu, L. Chen, Y.-S. Hu, *Adv. Funct. Mater.* **2021**, *31*, 2101475.

[4] W. Zuo, J. Qiu, X. Liu, F. Ren, H. Liu, H. He, C. Luo, J. Li, G. F. Ortiz, H. Duan, J. Liu, M.-S. Wang, Y. Li, R. Fu, Y. Yang, *Nat. Commun.* **2020**, *11*, 3544.

[5] a)T. Zhang, H. Ji, X. Hou, W. Ji, H. Fang, Z. Huang, G. Chen, T. Yang, M. Chu, S. Xu, Z. Chen, C. Wang, W. Yang, J. Yang, X. Ma, K. Sun, D. Chen, M. Tao, Y. Yang, J. Zheng, F. Pan, Y. Xiao, *Nano Energy* **2022**, *100*, 107482; b)H.-R. Yao, P.-F. Wang, Y. Gong, J. Zhang, X. Yu, L. Gu, C. OuYang, Y.-X. Yin, E. Hu, X.-Q. Yang, E. Stavitski, Y.-G. Guo, L.-J. Wan, *J. Am. Chem. Soc.* **2017**, *139*, 8440.

[6] a)D. Li, X. Ren, Q. Ai, Q. Sun, L. Zhu, Y. Liu, Z. Liang, R. Peng, P. Si, J. Lou, J. Feng, L. Ci, *Adv. Energy Mater.* **2018**, *8*, 1802386; b)Y. Xu, Y. Zhu, Y. Liu, C. Wang, *Adv. Energy Mater.* **2013**, *3*, 128.

[7] P.-F. Wang, H.-R. Yao, X.-Y. Liu, Y.-X. Yin, J.-N. Zhang, Y. Wen, X. Yu, L. Gu, Y.-G. Guo, *Sci. Adv.* **2018**, *4*, eaar6018.

[8] X.-Y. Du, Y. Meng, H. Yuan, D. Xiao, *Energy Storage Mater.* **2023**, *56*, 132.

[9] T. Yuan, S. Li, Y. Sun, J.-H. Wang, A.-J. Chen, Q. Zheng, Y. Zhang, L. Chen, G. Nam, H. Che, J. Yang, S. Zheng, Z.-F. Ma, M. Liu, *ACS Nano* **2022**, *16*, 18058.

[10] X. Zhang, Y.-N. Zhou, L. Yu, S.-Y. Zhang, X.-X. Xing, W. Wang, S. Xu, *Mater. Chem. Front.* **2021**, *5*, 5344.

[11] X. Meng, D. Zhang, Z. Zhao, Y. Li, S. Xu, L. Chen, X. Wang, S. Liu, Y. Wu, *J. Alloys Compd.* **2021**, *887*, 161366.

[12] N. Hong, K. Wu, Z. Peng, Z. Zhu, G. Jia, M. Wang, *J. Phys. Chem. C* **2020**, *124*, 22925.

[13] M. Leng, J. Bi, W. Wang, Z. Xing, W. Yan, X. Gao, J. Wang, R. Liu, *J. Alloys Compd.* **2020**, *816*, 152581.

[14] Q. Mao, C. Zhang, W. Yang, J. Yang, L. Sun, Y. Hao, X. Liu, *J. Alloys Compd.* **2019**, *794*, 509.

[15] Y. Xiao, P.-F. Wang, Y.-X. Yin, Y.-F. Zhu, Y.-B. Niu, X.-D. Zhang, J. Zhang, X. Yu, X.-D. Guo, B.-H. Zhong, Y.-G. Guo, *Adv. Mater.* **2018**, *30*, 1803765.

[16] P.-F. Wang, H.-R. Yao, X.-Y. Liu, J.-N. Zhang, L. Gu, X.-Q. Yu, Y.-X. Yin, Y.-G. Guo, *Adv. Mater.* **2017**, *29*, 1700210.
